# Kahweol Protects against Acetaminophen-Induced Hepatotoxicity in Mice through Inhibiting Oxidative Stress, Hepatocyte Death, and Inflammation

**DOI:** 10.1155/2022/8121124

**Published:** 2022-02-27

**Authors:** Jung-Yeon Kim, Jaechan Leem, Gyun Moo Kim

**Affiliations:** ^1^Department of Immunology, School of Medicine, Daegu Catholic University, Daegu 42472, Republic of Korea; ^2^Department of Emergency Medicine, School of Medicine, Daegu Catholic University, Daegu 42472, Republic of Korea

## Abstract

Acetaminophen (APAP) can cause acute liver failure, but treatment options are still limited. Kahweol is the main diterpene compound of coffee and possesses antioxidant and anti-inflammatory properties. Emerging evidence suggests that this natural diterpene exerts favorable effects on several inflammatory diseases. However, the action of kahweol on APAP toxicity has not been addressed. The purpose of this study was to explore whether kahweol has a protective activity against APAP-induced hepatotoxicity and to investigate the mechanism. Administration of kahweol reduced serum levels of liver injury indicators and ameliorated histological abnormalities in APAP-treated mice. Kahweol inhibited lipid peroxidation and nucleic acid oxidation with restoration of glutathione content and stimulation of nuclear factor erythroid-2-related factor 2-dependent cellular defense system. Hepatocyte death was also decreased by kahweol, which was associated with inhibition of endoplasmic reticulum (ER) stress. Moreover, kahweol reduced hepatic levels of inflammatory mediators, inhibited nuclear factor-*κ*B activation, and attenuated infiltration of neutrophils and macrophages. These findings suggest that kahweol has a protective activity against APAP-induced liver injury and this effect is related to the suppression of oxidative stress, hepatocyte death, ER stress, and inflammation.

## 1. Introduction

Acute liver failure (ALF) is a clinical condition characterized by the abrupt decline of liver function in patients without underlying liver disease, often accompanied by jaundice, coagulopathy, and hepatic encephalopathy [1]. The causes of ALF include viral hepatitis, drugs, and toxins [1]. Acetaminophen (APAP) is a widely used drug used to treat pain and fever. Although APAP is known to be safe when used at recommended doses, its overdose is considered the leading cause of ALF worldwide [2]. While this drug is primarily converted into nontoxic adducts and excreted in the urine, some are converted to form N-acetyl-p-benzoquinone imine (NAPQI) [3]. At recommended doses of APAP, the toxic metabolite can be neutralized via conjugation with endogenous glutathione (GSH). On the other hand, an overdose of APAP induces NAPQI accumulation along with GSH depletion, leading to the development of oxidative stress, hepatocellular death, and liver injury [3].

Because APAP-induced hepatotoxicity is associated with high mortality [2], much effort has been put into developing therapeutic approaches for this disease. However, to date, N-acetylcysteine (NAC) is the only treatment for APAP toxicity [4]. Administration of this drug at an early stage ameliorates APAP-induced hepatotoxicity by replenishing depleted GSH levels and preventing the accumulation of NAPQI. However, the clinical use of NAC is limited due to the narrow therapeutic window [5]. In addition, its efficacy is decreased when the treatment is started more than 8 h after poisoning [6]. Therefore, the development of novel drugs to treat APAP-induced hepatotoxicity is of great clinical importance.

Kahweol is a diterpene compound derived from coffee that inhibits tumor growth through its cytotoxic, antiproliferative, and antiangiogenic activities [7]. Emerging evidence suggests that the diterpene compound also displays antioxidant and anti-inflammatory effects in acute and chronic inflammatory diseases [7]. Recently, we showed that kahweol inhibited oxidative stress, apoptosis, and inflammatory responses to ameliorate cisplatin nephrotoxicity in mice [8]. Lee et al. also showed that kahweol exerted antioxidant effect to alleviate carbon tetrachloride- (CCl4-) induced hepatotoxicity [9]. In addition, the compound suppressed cytokine production in lipopolysaccharide- (LPS-) stimulated Kupffer cells and hepatocytes [10]. Besides inflammation, fibrotic processes were also attenuated by kahweol [11]. However, whether kahweol has a protective activity against APAP-induced hepatotoxicity has not been elucidated. Therefore, the present study evaluated the effects and underlying mechanism of the natural diterpene compound on APAP-induced hepatotoxicity.

## 2. Materials and Methods

### 2.1. Experimental Design

Twenty-four male C57BL/6N mice (7 weeks of age) were obtained from Hyosung Science (Daegu, South Korea) and were kept at a humidity of 40-60% and a temperature of 20 ± 2°C. All mice were acclimatized for 1 week and arbitrarily grouped to three groups: (a) control (*n* = 8), (b) APAP (*n* = 8), and (c) APAP combined with kahweol (APAP+Kah; *n* = 8). After 12 h of fasting, APAP (400 mg/kg; Sigma-Aldrich, St. Louis, MO) was administered intraperitoneally to the mice in the APAP and APAP+Kah groups. The APAP+Kah group received an intraperitoneal administration of kahweol (20 mg/kg; dissolved in DMSO; Abcam, Cambridge, MA) 1 h before APAP treatment. The same amount of DMSO was administered to the mice in the control group. All mice were sacrificed 24 h after APAP treatment, after which their blood and liver tissues were isolated for further analysis. The dosages of kahweol and APAP were determined based on previous studies [8, 12]. All experimental protocols were approved by the Institutional Animal Care and Use Committee of the Daegu Catholic University Medical Center (approval number: DCIAFCR-200507-05-Y, approval date: 7 May 2020).

### 2.2. Biochemical Analysis

Serum aspartate aminotransferase (AST) and alanine aminotransferase (ALT) activities were evaluated using a chemistry analyzer (Hitachi, Osaka, Japan). Hepatic malondialdehyde (MDA) levels were analyzed using a MDA measurement assay kit (Sigma-Aldrich). Hepatic amounts of GSH and oxidized GSH (GSSG) were evaluated using a glutathione detection assay kit (Abcam). Hepatic concentrations of tumor necrosis factor-*α* (TNF-*α*) and interleukin-6 (IL-6) were measured using ELISA kits (R&D Systems, Minneapolis, MN). All analyses were carried out following the manufacturers' protocols.

### 2.3. Histological Analysis and Immunohistochemistry (IHC)

After fixation, liver tissues were dehydrated, paraffin-embedded, and sectioned. The sections were stained with hematoxylin and eosin (H&E) and examined under a slide scanner (3DHISTECH, Budapest, Hungary). For IHC, the paraffin-embedded sections were deparaffinized. After rehydration and antigen retrieval, the sections were probed with primary antibodies for 4-hydroxynonenal (4-HNE; Abcam), CCAAT/enhancer-binding protein homologous protein (CHOP; Thermo Fisher Scientific, Waltham, MA), or F4/80 (Santa Cruz Biotechnology, Dallas, TX). After washing, the sections were incubated with horseradish peroxidase- (HRP-) conjugated secondary antibodies. Quantification of necrotic area and positively stained area was calculated using the i-Solution DT software (IMT i-Solution, Coquitlam, BC, Canada) in 5 randomized fields per sample. The number of F4/80-positive cells was assessed in 10 randomized fields per sample.

### 2.4. Immunofluorescence (IF) and TdT-Mediated dUTP Nick End Labeling (TUNEL) Staining

Sections were immunostained with a primary antibody for 8-hydroxy-2′-deoxyguanosine (8-OHdG; Santa Cruz Biotechnology) or Ly6B.2 (Abcam) and then incubated with an Alexa Fluor 488-coupled secondary antibody (Invitrogen, Carlsbad, CA). TUNEL staining was carried out using a TUNEL assay kit (TaKaRa, Tokyo, Japan) following the manufacturer's protocol. Images were acquired using a confocal microscope (Nikon, Tokyo, Japan). DAPI was used for nuclear staining. Positively stained cells were examined in 10 randomized fields per sample.

### 2.5. Western Blotting

Liver tissues were homogenized using a lysis buffer (Sigma-Aldrich) to isolate total proteins, which were subjected to polyacrylamide gel electrophoresis. Separated proteins were transferred to nitrocellulose membranes, and the membranes were incubated with primary antibodies for nuclear factor erythroid-2-related factor 2 (Nrf2; Abcam), heme oxygenase-1 (HO-1: Invitrogen), spliced X-box binding protein 1 (XBP1s; Cell Signaling, Danvers, MA), eukaryotic initiation factor 2*α* (eIF2*α*; Cell Signaling), p-eIF2*α* (Cell Signaling), CHOP (Thermo Fisher Scientific), nuclear factor-*κ*B p65 (NF-*κ*B p65; Cell Signaling), p-NF-*κ*B p65 (Cell Signaling), glyceraldehyde-3-phosphate dehydrogenase (GAPDH; Cell Signaling), and lamin B1. After washing, the membranes were probed with HRP-coupled secondary antibodies. Signal intensities were analyzed using an image analyzer (Thermo Fisher Scientific) and ImageJ software.

### 2.6. qPCR Analysis

Total RNA was isolated from liver tissues using TRIzol reagent (Sigma-Aldrich) and reversely transcribed into cDNA using the High-Capacity cDNA Reverse Transcription Kit (Applied Biosystems, Foster City, CA). Real-time RT-PCR was conducted in a Thermal Cycler Dice Real Time System III (TaKaRa). Primers are shown in Table 1. Data were calculated using the 2^-*ΔΔ*CT^ method and normalized to GAPDH levels.

### 2.7. Statistical Analysis

Data are presented as mean ± SEM. Statistical significance between the groups was assessed using one-way ANOVA and Turkey's *post hoc* test. *p* values less than 0.05 were considered statistically significant.

## 3. Results

### 3.1. Kahweol Alleviated APAP-Induced Acute Liver Injury

**Figure 1:**
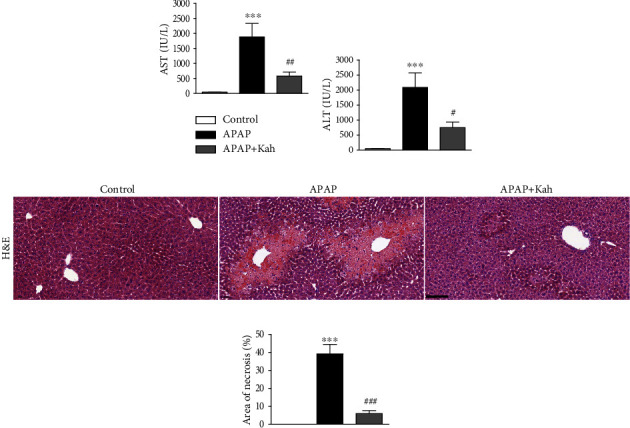
Effects of kahweol on serum AST and ALT levels and histological alterations in APAP-treated mice. (a) Serum AST levels. (b) Serum ALT levels. (c) H&E staining. Scale bar = 100 *μ*m. (d) Percentage of necrotic area. *n* = 8 per group. ^∗∗∗^*p* < 0.001 vs. control. ^#^*p* < 0.05, ^##^*p* < 0.01, and ^###^*p* < 0.001 vs. APAP.

AST and ALT are established indicators of liver damage [13]. Serum AST and ALT levels were elevated after APAP treatment (Figures 1(a) and 1(b)). However, kahweol significantly decreased the elevation of liver injury indicators (Figures 1(a) and 1(b)). In addition, H&E staining showed a larger area of necrosis in the APAP group than in the control group (Figures 1(c) and 1(d)). Kahweol also decreased the necrotic area in the APAP-treated mice (Figures 1(c) and 1(d)).

### 3.2. Kahweol Attenuated APAP-Induced Oxidative Stress

**Figure 2:**
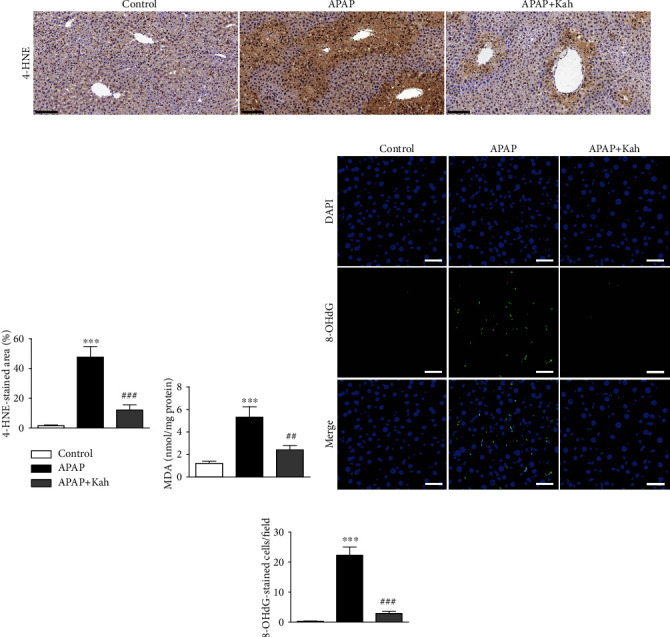
Effects of kahweol on lipid peroxidation and DNA oxidation in APAP-treated mice. (a) IHC staining for 4-HNE. Scale bar = 100 *μ*m. (b) Percentage of 4-HNE-stained area. (c) Hepatic MDA levels. (d) IF staining for 8-OHdG (green). Nuclei were counterstained with DAPI (blue). Scale bar = 40 *μ*m. (e) Number of 8-OHdG-stained cells per field. *n* = 8 per group. ^∗∗∗^*p* < 0.001 vs. control. ^##^*p* < 0.01 and ^###^*p* < 0.001 vs. APAP.

Oxidative stress is a critical pathological factor in APAP-induced hepatotoxicity [14]. To determine the effect of kahweol on oxidative stress, we analyzed hepatic levels of 4-HNE and MDA. These molecules are well-known products of lipid peroxidation [15]. IHC staining showed that the percentage of 4-HNE-stained area was increased after APAP treatment (Figures 2(a) and 2(b)). APAP treatment also increased the amount of MDA (Figure 2(c)). However, kahweol significantly attenuated these changes (Figures 2(a)–2(c)). In addition, APAP treatment increased the number of cells stained with 8-OHdG, a marker of nucleotide oxidation [16], which was also significantly decreased by kahweol (Figures 2(d) and 2(e)).

**Figure 3:**
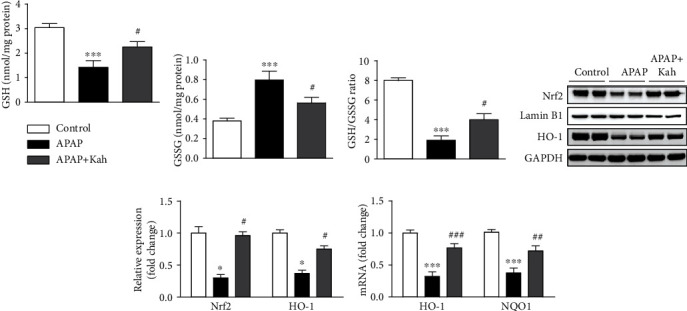
Effects of kahweol on GSH levels, GSSG levels, GSH/GSSG ratios, and Nrf2 pathway in APAP-treated mice. (a) Hepatic GSH levels. (b) Hepatic GSSG levels. (c) GSH/GSSG ratios. (d) Western blotting of Nrf2 and HO-1. (e) Quantification of western blot data for Nrf2 and HO-1. (f) Hepatic mRNA levels of HO-1 and NQO1. *n* = 8 per group. ^∗^*p* < 0.05 and ^∗∗∗^*p* < 0.001 vs. control. ^#^*p* < 0.05, ^##^*p* < 0.01, and ^###^*p* < 0.001 vs. APAP.

We also observed that GSH levels were reduced after APAP treatment but were significantly restored by kahweol (Figure 3(a)). The increase in GSSG levels and the decrease in the GSH/GSSG ratios after APAP treatment were also significantly reversed by kahweol (Figures 3(b) and 3(c)). Given that Nrf2 is known to modulate the cellular antioxidant defense system [17], we next assessed the effect of the compound on Nrf2. Nuclear Nrf2 levels were reduced after APAP treatment (Figures 3(d) and 3(e)). Consistently, protein and mRNA expression of heme oxygenase-1, a target gene of Nrf2, was decreased after APAP treatment (Figures 3(d)–3(f)). In addition, APAP treatment also decreased NAD(P)H:quinone oxidoreductase 1 (NQO1) expression (Figure 3(f)). However, APAP-induced inhibition of the Nrf2 pathway was significantly attenuated by kahweol (Figures 3(d)–3(f)).

### 3.3. Kahweol Inhibited APAP-Induced Hepatocyte Death and Endoplasmic Reticulum (ER) Stress

**Figure 4:**
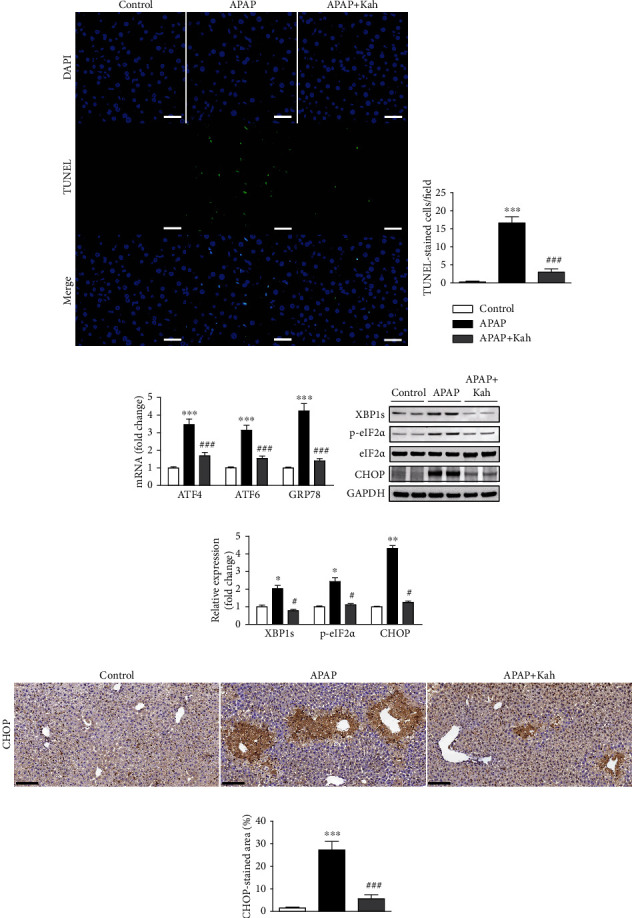
Effects of kahweol on hepatocyte death and ER stress in APAP-treated mice. (a) TUNEL staining. Nuclei were counterstained with DAPI (blue). Scale bar = 40 *μ*m. (b) Number of TUNEL-stained cells per field. (c) Hepatic mRNA levels of ATF4, ATF6, and GRP78. (d) Western blotting of XBP1s, p-eIF2*α*, and CHOP in liver tissues. (e) Quantification of western blot data for XBP1s, p-eIF2*α*, and CHOP. (f) IHC staining for CHOP. Scale bar = 100 *μ*m. (g) Percentage of CHOP-stained area. *n* = 8 per group. ^∗^*p* < 0.05, ^∗∗^*p* < 0.01, and ^∗∗∗^*p* < 0.001 vs. control. ^#^*p* < 0.05 and ^###^*p* < 0.001 vs. APAP.

Hepatocyte death plays an important role in APAP-induced hepatotoxicity [18]. To assess the effect of kahweol on hepatocyte death, we carried out TUNEL staining of liver sections. Number of positive cells was elevated after APAP treatment (Figures 4(a) and 4(b)). However, kahweol significantly reduced hepatocyte death in APAP-treated mice (Figures 4(a) and 4(b)).

Emerging evidence suggests that ER stress can induce hepatocyte death in APAP-induced hepatotoxicity [18, 19]. Thus, we next examined the effect of kahweol on ER stress. APAP treatment increased mRNA levels of activating transcription factor 4 (ATF4), ATF6, and glucose-regulated protein 78 (GRP78) (Figure 4(c)). Protein levels of XBP1s, p-eIF2*α*, and CHOP were also increased after APAP treatment (Figures 4(d) and 4(e)). However, kahweol significantly decreased the expression of these ER stress markers (Figures 4(c)–4(e)). In addition, IHC staining revealed that APAP treatment increased the percentage of CHOP-stained area, which was significantly reduced by kahweol (Figures 4(f) and 4(g)).

### 3.4. Kahweol Alleviated APAP-Induced Inflammation

**Figure 5:**
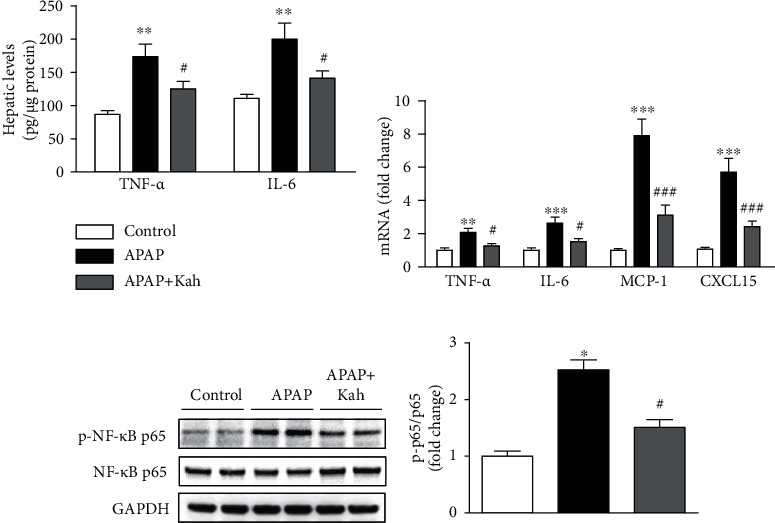
Effects of kahweol on production of inflammatory mediators and NF-*κ*B activation in APAP-treated mice. (a) Hepatic levels of TNF-*α* and IL-6 proteins. (b) Hepatic mRNA levels of TNF-*α*, IL-6, MCP-1, and CXCL15. (c) Western blotting of p-NF-*κ*B p65. (d) Quantification of western blot data for p-NF-*κ*B p65. *n* = 8 per group. ^∗^*p* < 0.05, ^∗∗^*p* < 0.01, and ^∗∗∗^*p* < 0.001 vs. control. ^#^*p* < 0.05 and ^###^*p* < 0.001 vs. APAP.

Inflammation is involved in the pathophysiology of APAP-induced hepatotoxicity [5]. APAP treatment increased hepatic concentrations of TNF-*α* and IL-6 proteins (Figure 5(a)). In addition, mRNA levels of TNF-*α*, IL-6, monocyte chemoattractant protein-1 (MCP-1), and C-X-C motif chemokine 15 (CXCL15) were also elevated after APAP treatment (Figure 5(b)). However, the increase was significantly attenuated by kahweol (Figures 5(a) and 5(b)). Moreover, protein expression of NF-*κ*B p65 was increased after APAP treatment, which was significantly reduced by kahweol, indicating that kahweol inhibited NF-*κ*B activation (Figures 5(c) and 5(d)).

**Figure 6:**
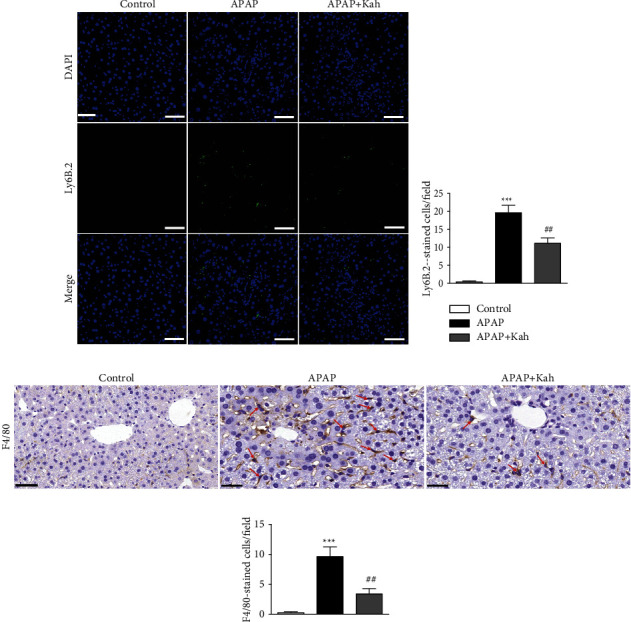
Effects of kahweol on infiltration of neutrophils and macrophages in APAP-treated mice. (a) IF staining for Ly6B.2 (green). Nuclei were counterstained with DAPI (blue). Scale bar = 100 *μ*m. (b) Number of Ly6B.2-stained cells per field. (c) IHC staining for F4/80. Positive cells were indicated using red arrows. Scale bar = 30 *μ*m. (d) Number of F4/80-stained cells. *n* = 8 per group. ^∗∗∗^*p* < 0.001 vs. control. ^##^*p* < 0.01 vs. APAP.

It has been known that inflammatory cells infiltrate the liver and play an important role in APAP-induced hepatotoxicity [20, 21]. Thus, we next examined whether kahweol can attenuate the infiltration of neutrophils and macrophages in the APAP-treated mice. IF staining for Ly6B.2, a neutrophil marker [22], showed that the number of positively stained cells was increased after APAP treatment (Figures 6(a) and 6(b)). However, kahweol significantly reduced the infiltration of Ly6B.2-positive cells in the APAP-treated mice (Figures 6(a) and 6(b)). IHC staining revealed that the increase in the number of F4/80-positive cells after APAP treatment was also significantly decreased by kahweol (Figures 6(c) and 6(d)).

## 4. Discussion

APAP overdose is the leading cause of ALF, but the therapeutic options for it are still limited [2]. In this study, we showed that kahweol protected against APAP-induced acute hepatotoxicity via inhibition of oxidative stress, hepatocyte death, ER stress, and inflammation.

AST and ALT are enzymes located mainly in hepatocytes. When the liver is injured, these enzymes are released from damaged hepatocytes into the bloodstream. AST and ALT are sensitive indicators of hepatocellular damage [23]. In this study, kahweol decreased serum levels of the liver injury indicators in the APAP-treated mice. Histological examination of the liver sections showed that APAP-induced hepatic necrosis was significantly alleviated by the compound. A rodent model of CCl4-induced acute hepatotoxicity is widely used to explore new therapeutic agents for ALF [24, 25]. Similar to our findings, a recent study reported that kahweol ameliorated CCl4-induced liver injury [9]. Thus, the hepatoprotective effect of kahweol may be applied to various types of ALF.

Although the underlying mechanisms for APAP toxicity remain incompletely understood, oxidative stress is considered a critical process in its pathogenesis [14]. At recommended doses of APAP, NAPQI is detoxified via conjugation with GSH. However, at toxic doses of APAP, excessive NAPQI causes hepatic GSH depletion, leading to increased oxidative stress [3]. In the present study, we analyzed the major products of lipid peroxidation (4-HNE and MDA) and DNA oxidation (8-OHdG) to evaluate oxidative stress. Kahweol significantly reduced the increased amounts of these oxidative products with restoration of GSH levels in APAP-treated mice, indicating that kahweol exhibits antioxidant activity in APAP-induced hepatotoxicity. Similar to these findings, previous studies have reported that kahweol ameliorated several inflammatory diseases via its antioxidant activity [8, 9]. *In vitro* studies have also shown the antioxidant effect of kahweol in dopaminergic neurons [26] and pancreatic *β*-cells [27]. Antioxidant enzymes play a critical role in protecting the liver from APAP-induced oxidative stress [5]. In particular, accumulating evidence suggests the Nrf2 activation could be served as a useful therapeutic approach for APAP-induced hepatotoxicity [28–30]. Nrf2 regulates antioxidant defense enzymes to inhibit oxidative stress [17]. In this study, APAP treatment suppressed nuclear translocation of Nrf2 and downregulated its target genes in the liver. However, kahweol significantly activated the Nrf2 pathway in APAP-treated mice. Therefore, our data suggest that kahweol attenuates APAP-induced oxidative stress through restoration of GSH content and activation of the Nrf2-mediated antioxidant system.

In APAP-induced hepatotoxicity, hepatocyte death is primarily mediated by oxidative stress and plays an important role in its pathophysiology [18]. Among the modes of cell death, necrosis is considered the major form of hepatocyte death in APAP toxicity, whereas the role of apoptosis remains controversial [19, 31]. In this study, we carried out TUNEL staining to detect hepatocyte death on liver sections. TUNEL staining is widely used to identify apoptotic cells, but it is not specific for apoptosis and can also detect necrotic cells [32]. Administration of kahweol decreased the number of cells with TUNEL staining, indicating that kahweol protected hepatocytes from APAP-induced cell death. Similar to our findings, several studies have reported the cytoprotective effects of kahweol [26, 27]. ER is an intracellular organelle where proteins are folded, assembled, and posttranslationally modified. ER stress response is triggered by various conditions that disturb protein folding in the ER [33]. It has been known that ER stress is detected in a broad range of liver diseases [33]. Emerging evidence suggests that ER stress induces hepatocyte death in APAP-induced hepatotoxicity [18, 19]. CHOP is a transcription factor that modulates cell death in ER stress and is upregulated during APAP-induced hepatotoxicity [19]. CHOP knockout mice were resistant to hepatocyte necrosis and displayed increased survival compared to control mice after APAP treatment. In the present study, the occurrence of ER stress by APAP was demonstrated by increased expression of key molecules (ATF4, ATF6, GRP78, XBP1s, and p-eIF2*α*) of the ER stress response. Importantly, CHOP expression was also increased by APAP treatment. However, the APAP-induced activation of ER stress response with CHOP upregulation was significantly alleviated by kahweol. These results suggest that the diterpene compound alleviates APAP-induced hepatocyte death and that this cytoprotective effect is associated with suppression of ER stress.

Rupture of cells during necrosis causes of the release of various mediators from necrotic cells to extracellular space, promoting the production of proinflammatory mediators in immune cells [5]. This process enhances the infiltration of inflammatory cells into the damaged liver, exacerbating hepatocyte death. In this study, APAP treatment increased production of cytokines (TNF-*α* and IL-6) and chemokines (MCP-1 and CXCL15) and accumulation of neutrophils and macrophages. However, kahweol significantly inhibited the inflammatory responses induced by APAP. Furthermore, the anti-inflammatory effect of kahweol was accompanied by inhibition of the NF-*κ*B pathway. NF-*κ*B is a key player that modulates expression of proinflammatory cytokines [34]. Several studies have shown the involvement of the NF-*κ*B signaling pathway in APAP-induced production of proinflammatory mediators [35, 36]. Altogether, our data showed that kahweol attenuated cytokine and chemokine production and inflammatory cell infiltration with concomitant suppression of NF-*κ*B activation in APAP-induced hepatotoxicity. Seo et al. showed that the compound suppressed the NF-*κ*B pathway to decrease cytokine production in LPS-treated mouse primary Kupffer cells and hepatocytes [10]. Recently, we also showed that kahweol attenuated proinflammatory cytokine production and inflammatory cell infiltration with inhibition of the NF-*κ*B pathway in an animal model of cisplatin nephrotoxicity [8].

In conclusion, our data demonstrated that kahweol protects against APAP-induced liver injury by activating the antioxidant system, inhibiting ER stress-induced hepatocyte death, and attenuating NF-*κ*B-mediated inflammation. These results suggest that kahweol could be a potential agent for treatment of APAP-induced liver injury.

## Figures and Tables

**Table 1:** List of primers used in this study.

Gene	Primer sequence (5′→3 ′)	Accession No.
HO-1	F: TCAAGGCCTCAGACAAATCC R: ACAACCAGTGAGTGGAGCCT	NM_010442
NQO1	F: AATGGGCCAGTACAATCAGG R: CCAGCCCTAAGGATCTCTCC	NM_008706
ATF4	F: GAGCTTCCTGAACAGCGAAGTG R: TGGCCACCTCCAGATAGTCATC	NM_009716
ATF6	F: CCCAAGCTCTCCGCATAGTC R: TAAAATGCCCCATAACTGACCAA	NM_001081304
GRP78	F: TGGTATTCTCCGAGTGACAGC R: AGTCTTCAATGTCCGCATCC	NM_001163434
TNF-*α*	F: GACGTGGAACTGGCAGAAGAG R: CCGCCTGGAGTTCTGGAA	NM_013693
IL-6	F: CCAGAGATACAAAGAAATGATGG R: ACTCCAGAAGACCAGAGGAAAT	NM_031168
MCP-1	F: TAAAAACCTGGATCGGAACCAA R: GCATTAGCTTCAGATTTACGGGT	NM_011333
CXCL15	F: ATGGCTGCTCAAGGCTGGTC R: AGGCTTTTCATGCTCAACACTAT	NM_011339
GAPDH	F: ACTCCACTCACGGCAAATTC R: TCTCCATGGTGGTGAAGACA	NM_001289726

## Data Availability

The data supporting the findings of this study are available within the article.
